# Effect of Facial Cosmetic Acupuncture on Facial Elasticity: An Open-Label, Single-Arm Pilot Study

**DOI:** 10.1155/2013/424313

**Published:** 2013-07-28

**Authors:** Younghee Yun, Sehyun Kim, Minhee Kim, KyuSeok Kim, Jeong-Su Park, Inhwa Choi

**Affiliations:** ^1^Department of Dermatology of Korean Medicine, College of Korean Medicine, Kyung Hee University, No. 149 Sangil-dong, Gangdong-gu, Seoul 134-727, Republic of Korea; ^2^Graduate School of East-West Medical Science, Kyung Hee University, Yongin, Gyeonggi-Do 446-701, Republic of Korea; ^3^Kyung Hee Center for Clinical Research and Drug Development, Kyung Hee University, 1 Hoegidong, Dongdaemungu, Seoul 130-701, Republic of Korea; ^4^Department of Preventive Medicine, Graduate School of Korean Medicine, Kyung Hee University, Seoul 130-701, Republic of Korea

## Abstract

*Background*. The use of acupuncture for cosmetic purposes has gained popularity worldwide. Facial cosmetic acupuncture (FCA) is applied to the head, face, and neck. However, little evidence supports the efficacy and safety of FCA. We hypothesized that FCA affects facial elasticity by restoring resting mimetic muscle tone through the insertion of needles into the muscles of the head, face, and neck. *Methods*. This open-label, single-arm pilot study was implemented at Kyung Hee University Hospital at Gangdong from August through September 2011. Participants were women aged 40 to 59 years with a Glogau photoaging scale III. Participants received five treatment sessions over three weeks. Participants were measured before and after FCA. The primary outcome was the Moire topography criteria. The secondary outcome was a patient-oriented self-assessment scale of facial elasticity. *Results*. Among 50 women screened, 28 were eligible and 27 completed the five FCA treatment sessions. A significant improvement after FCA treatment was evident according to mean change in Moire topography criteria (from 1.70 ± 0.724 to 2.26 ± 1.059, *P* < 0.0001). The most common adverse event was mild bruising at the needle site. *Conclusions*. In this pilot study, FCA showed promising results as a therapy for facial elasticity. However, further large-scale trials with a controlled design and objective measurements are needed.

## 1. Introduction

With extended life expectancy, beauty and skin health are important factors in perceived quality of life. Currently, numerous interventions are offered for skin rejuvenation and anti-skin aging including treatments for facial wrinkles, facial muscle tone, and elasticity. Recently, cosmetic acupuncture has been introduced as an intervention for skin rejuvenation [1].

Facial cosmetic acupuncture (FCA) is the use of acupuncture on the head, face, and neck for cosmetic purposes. Several different types of FCA are currently practiced, and many possible mechanisms underlying these techniques have been proposed, including increasing or balancing qi, balancing internal Zang Fu organs, increasing blood flow by inserting needles at certain acupoints [2], and increasing muscle tone [3]. 

However, little evidence addresses the efficacy and safety of FCA. A recent case report describes the increased water and oil content of facial skin after FCA [4]; otherwise, there is only an introductory [1, 2] or non-English article [3]. To explore whether FCA has effects on facial elasticity, we designed an open-label, single-arm pilot study using the most frequently practiced FCA technique in Korea.

## 2. Participants and Methods

### 2.1. Ethics Approval

This study was performed in accordance with the International Committee on Harmonization Good Clinical Practice guidelines and the revised version of the Declaration of Helsinki. The trial protocol was approved by the Institutional Review Board of Kyung Hee University Hospital at Gangdong (KHNMC-OH-IRB 2011-007). Written informed consent was obtained from all participants prior to enrollment, and participants were given ample time to decide about participating before signing the consent form.

### 2.2. Participant Recruitment and Inclusion/Exclusion Criteria

Participants were recruited by advertisements on bulletin boards at Kyung Hee University Hospital at Gangdong. Included were (a) women; (b) aged 40 to 59 years; (c) with a Glogau photoaging scale III [5]. We excluded individuals who (a) had dermabrasion, deep skin peels, laser resurfacing (ablative or nonablative), botulinum toxin, filler injection, or topical steroid treatment within the 6 months immediately prior to study entry; (b) had obvious skin disease or a history of chronic skin disease; (c) had a keloidal or hypertrophic scar tendency; or (d) were pregnant or breastfeeding. No other treatment for facial elasticity was permitted during the study period.

### 2.3. Study Protocol

This study was an open-label, single-arm pilot study at Kyung Hee University Hospital at Gangdong from August through September 2011. Five sessions of FCA treatment were given over three weeks. All participants received FCA twice a week for the first two weeks, then once a week for the last week, with three to four days between sessions. Participants were assessed based on changes in the Moire topography criteria [6]. 

### 2.4. Acupuncture Procedure

**Figure 1:**
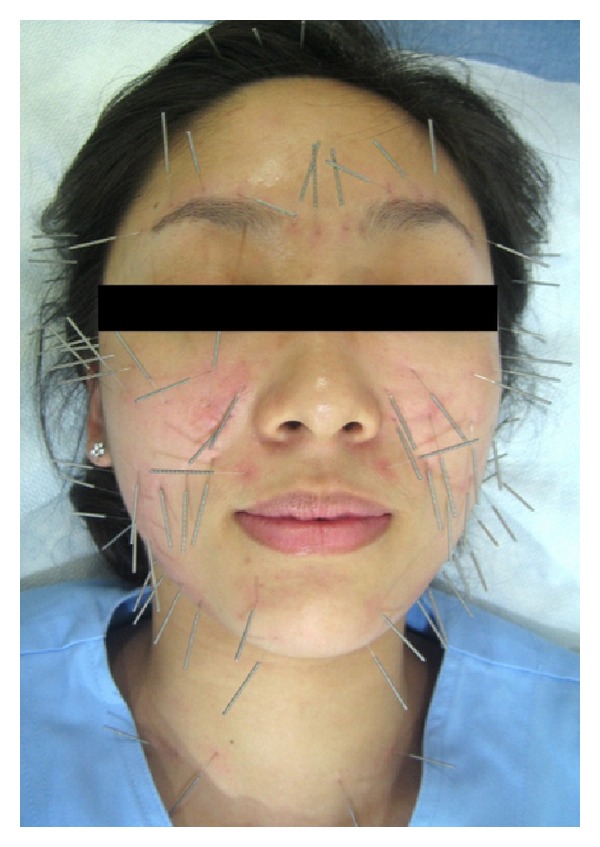
Facial cosmetic acupuncture applied in this study.

Acupuncture was applied (Figure 1) according to the Standards for Reporting Interventions in Clinical Trials of Acupuncture (STRICTA) [7].

**Figure 2:**
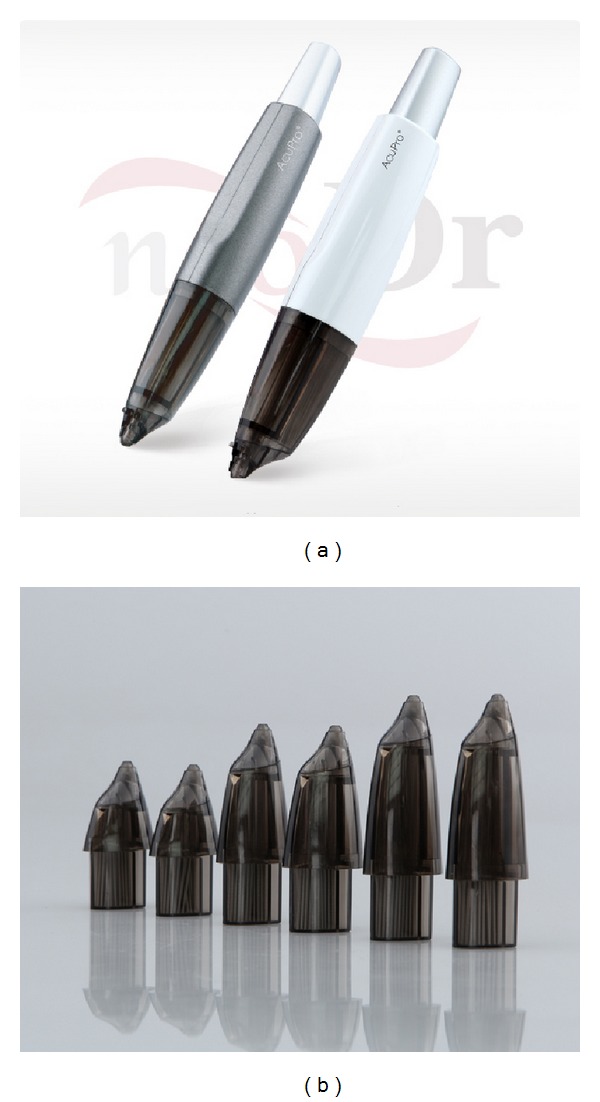
AcuPro and stainless steel fine needles used in this study.

1. Acupuncture rationale
  - A single practitioner inserted acupuncture needles into muscles of the face, head, and neck.
  - All participants received the same FCA treatment at every treatment session.
2. Needling details
  - The total number of insertions per treatment ranged from approximately 100 to 110.
  - The practitioner inserted acupuncture needles at the insertion, origin, belly and/or margin of
    - head muscles including the temporalis and epicranial aponeurosis;
    - neck muscles including the sternocleidomastoid;
    - upper facial muscles including the frontalis, procerus, corrugator supercilii, and orbicularis oculi;
    - midfacial muscles including the auricularis, nasalis, levator labii superioris alaeque nasi, levator labii superioris, zygomaticus minor, and zygomaticus major;
    - lower facial muscles including the orbicularis oris, risorius, depressor labii inferioris, depressor anguli oris, mentalis, and platysma.
  - The depth of needle insertion varied with skin thickness and subcutaneous fatty tissue at the insertion site.
  - The practitioner did not use any specific needling technique. However, the practitioner tried to insert needles into the contraction of muscles fibers over the muscle insertions, origins, bellies, and/or margins of muscles.
  - Needles were retained for ten minutes.
  - The practitioner used an acupuncture treatment aid, AcuPro (NEO Dr.), and stainless steel fine needles (0.2 × 15 mm, 0.25 × 30 mm) to reduce pain and to shorten treatment time (Figure 2).
3. Treatment regimen
  - All participants received five sessions FCA over the 3-week treatment period.
  - All participants received FCA twice a week for the first two weeks, and then once a week for the last week.
4. Other components of treatment
  - No other treatments were given and participants were asked not to receive any other treatment for facial elasticity during the study period.
  - All participants received FCA with an interval of three to four days between sessions.

### 2.5. Outcome Measurements

Outcomes were measured before and after the five sessions of FCA.

### 2.6. Primary Outcome

**Figure 3:**
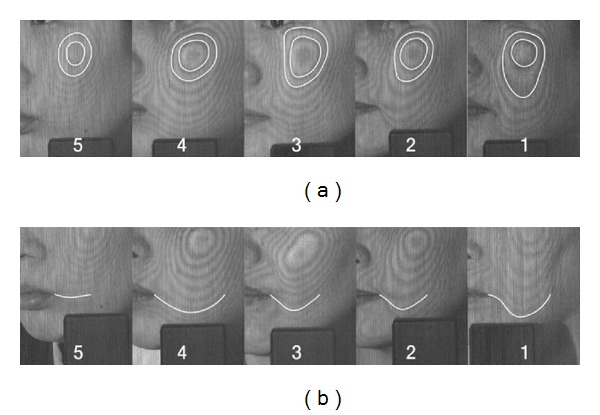
Criteria for evaluating Moire topography.

The primary outcome was a change in the Moire topography criteria after treatment compared with baseline. We generated contour lines on the face using a Moire topography system and took pictures with a digital camera Ixus750 (Canon, Tokyo, Japan). A single independent evaluator read the contour lines near the cheek and the perioral region in the printed digital image and graded the images based on the Moire topography criteria (Figure 3) [6].

### 2.7. Secondary Outcomes

A patient-oriented self-assessment scale of facial elasticity was performed with the same frequency as the primary measurements. Participants assessed their degrees of the facial elasticity using a 10 cm vertical line visual analog scale (VAS). The scale was marked at the top with “most severe condition,” with the bottom labeled “fine condition.”

### 2.8. Safety

The Institutional Review Board of Kyung Hee University Hospital at Gangdong reviewed the protocol, monitored patient safety, and investigated any adverse events independently of the investigators.

### 2.9. Statistical Analysis

All primary analyses were based on an intention-to-treat (ITT) population. End-of-study analyses were performed using the last observation carried forward for participants who did not complete the study. Patient characteristics were summarized using descriptive statistics. The nonparametric Wilcoxon signed-rank test was used for assessing clinical improvement. SPSS 15.0 for Windows (SPSS Inc., Chicago, IL, USA) was used for data management and statistical analysis. A *P* value less than 0.05 was considered statistically significant.

### 2.10. Quality Control

Before starting the trial, the acupuncture practitioner was trained and had been administering FCA at a clinic of Kyung Hee University Hospital at Gangdong for over a year. The investigator who assessed the outcomes received thorough training in assessing Moire topography. 

## 3. Results

### 3.1. Participants

**Figure 4:**
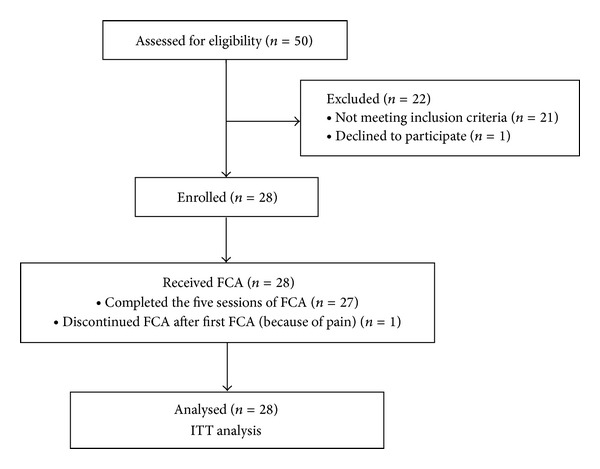
Progression of participants through the study.

Of 50 participants screened, 28 were eligible for the study, 27 completed the five sessions of FCA treatment, and one dropped out because of pain after the first FCA treatment. The mean age was 50.04 ± 6.07 (range: 40–59) years, and all participants were Asian females with a Glogau photoaging scale III (Figure 4). 

### 3.2. Primary Outcome

The primary outcome was mean change in Moire topography criteria from baseline to the end of the study in the ITT population. The Moire topography changed significantly (*P* = 0.0001) after FCA treatment (Table 1). Of the 27 participants who underwent all five sessions, 12 exhibited no change, while 15 showed a positive, single-level improvement (Table 2). 

### 3.3. Secondary Outcomes

Mean changes in a patient self-assessment of skin elasticity showed no significant differences (Table 3).

### 3.4. Safety Evaluation

The most commonly reported adverse event that was clearly attributable to FCA treatment was mild bruising (20/140 treatment sessions; 14.28%) at the needle site. Only one participant dropped out because of pain. No adverse events of scarring, nerve damage, or lengthy recovery periods were observed.

## 4. Discussion

This clinical open-label, single-arm pilot study investigated the efficacy and safety of FCA on facial elasticity. FCA has been increasing in use and popularity but few introductory articles [1, 2] were available until Donoyama et al. reported in 2012 on increased water and oil content for facial skin after cosmetic acupuncture [4].

Several different types of FCA are practiced. Recently, in Korea, clinicians have used FCA to enhance facial elasticity by restoring resting mimetic muscle tone by inserting needles into head, face, and neck muscles. Louarn et al. [8] conducted an MRI study on changes in the contour of facial mimetic muscles in patients of different ages. They found that facial mimetic muscles gradually straighten and shorten with age as a result of increased resting muscle tone. Based on these findings, we hypothesized that FCA could be used to improve facial elasticity with needles inserted into the muscles of the head, face, and neck, resulting in restored muscle tone.

Different methods for measuring facial elasticity range from manual examination to direct visualization. Moire topography is an optical measurement that does not require direct contact and allows high-precision visualization of facial shape in three dimensions, similar to a contour map [9]. Moire topography is used in studies of facial palsy, zygomatic fractures [10], facial morphology, and facial plastic surgery [11]. The Moire topography criteria were developed by Ahn et al. [6] for measuring facial elasticity. Moire topography criteria show a very high correlation with age and the Cutometer, which evaluates skin elasticity.

In this study, we found that participants who underwent five FCA treatment sessions showed an improvement of about 0.5 by Moire topography. FCA also improved scores on a patient self-assessment of elasticity, but the changes were not significant. These results suggested that FCA improved facial elasticity in women aged 40 and 59 years with a Glogau photoaging scale III.

This study had several limitations. It is an open-label, single-arm pilot design. The sample size was small with no control group, and the trial duration was short compared to the actual clinical environment. For example, in the Cosmetic/Derma Clinic of Kyung Hee University Hospital at Gangdong, an FCA treatment course is generally eight treatment sessions over 4 weeks. The Moire topography criteria are an ordinal scale with wide intervals. The scale might not detect small changes and is highly dependent on the evaluator's judgment.

However, in spite of these limitations, this study could be helpful in providing clinicians with procedural details about FCA and could be the basis of future investigations aimed at elucidating the possible mechanisms of FCA including restoration of resting mimetic muscle tone. A larger study with a controlled design using different objective outcomes measure could be warranted.

## Figures and Tables

**Table 1:** Mean change in Moire topography.

	Before FCA (*n* = 28)	After FCA (*n* = 28)	*P* value
Moire topography criteria	1.70 ± 0.724	2.26 ± 1.059	0.0001*

Data are mean ± standard deviation of percent change (95% confidence interval).

*Statistically significant difference, *P* < 0.05.

**Table 2:** Changes in Moire topography for participants who completed the study.

Negative change after FCA (*n*)	0
No change after FCA (*n*)	12
Single level improvement after FCA (*n*)	15

Total (*n*)	27

**Table 3:** Mean change in patient self-assessment of skin elasticity.

	Before FCA (*n* = 28)	After FCA (*n* = 28)	*P* value
Patient self-assessment Elasticity scale	6.15 ± 1.562	4.81 ± 1.942	0.006

Data are mean ± standard deviation of percent change (95% confidence interval).
